# Microglial morphology in Alzheimer’s disease and after Aβ immunotherapy

**DOI:** 10.1038/s41598-021-95535-0

**Published:** 2021-08-05

**Authors:** Diana K. Franco-Bocanegra, Yamina Gourari, Ciaran McAuley, David S. Chatelet, David A. Johnston, James A. R. Nicoll, Delphine Boche

**Affiliations:** 1 Clinical Neurosciences, Clinical and Experimental Sciences School, Faculty of Medicine, University of Southampton, Southampton, UK; 2 Biomedical Imaging Unit, Southampton General Hospital, University of Southampton, Southampton, UK; 3 Department of Cellular Pathology, University Hospital Southampton NHS Foundation Trust, Southampton, UK

**Keywords:** Microglia, Innate immunity, Alzheimer's disease

## Abstract

Microglia are the brain immune cells and their function is highly dependent on cell motility. It was hypothesised that morphological variability leads to differences in motility, ultimately impacting on the microglial function. Here, we assessed microglial morphology in 32 controls, 44 Alzheimer’s disease (AD) cases and 16 AD cases from patients immunised against Aβ42 (iAD) using 2D and 3D approaches. Our 2D assessment showed an increased number of microglia in iAD vs. AD (*P* = 0.032) and controls (*P* = 0.018). Ramified microglia were fewer in AD vs. controls (*P* = 0.041) but increased in iAD compared to AD (*P* < 0.001) and controls (*P* = 0.006). 3D reconstructions highlighted larger cell bodies in AD vs. controls (*P* = 0.049) and increased total process length in iAD vs. AD (*P* = 0.032), with negative correlations detected for pan-Aβ load with total process length (*P* < 0.001) in AD and number of primary processes (*P* = 0.043) in iAD. In summary, reactive/amoeboid microglia are the most represented population in the aged human brain. AD does not affect the number of microglia, but the ramified population is decreased adopting a more reactive morphology. Aβ removal by immunotherapy leads to increased ramified microglia, implying that the cells retain plasticity in an aged disease brain meriting further investigation.

## Introduction

Microglia are a key component of the resident immune system of the brain. They are, to some extent, similar to macrophages in their expression profile and in their behaviour and functions. Despite this resemblance, microglia have a different origin, since they do not derive from the bone marrow, but originate in the embryonic yolk sac and their precursors migrate directly to the central nervous system early in the development^1^. The characteristic microglial morphology consists of a distinct cell body (soma), from which elongated ramified processes are projected. However, the number, length and complexity of branching of the processes can vary widely particularly in reactive situations (e.g. insults, injury, disease), with variations in cell body size and shape also reported^2–4^. It has been hypothesised that the morphological variability leads to differences in motility and ultimately impacts on the microglial function^5,6^.

Evidence suggests microglial dysfunction has an important role in the development of Alzheimer’s disease (AD). In particular, most of the genes identified by genome wide association studies are expressed by microglia. Changes in microglial morphology have been observed in mouse models of AD. For example, in CRND8 mice which carry a mutated form of the human amyloid precursor protein gene, microglia in the proximity of Aβ plaques were less ramified compared to microglia distant from Aβ plaques, as well as compared to microglia from wild-type mice^7^. Evidence from two human studies including respectively 7 and 8 AD cases suggest morphological changes of microglia in AD^8,9^. The aim of the present study was to assess in detail and in a quantitative manner in largest cohorts of AD and control brains, several features of the microglial morphology imaged from human brain and compare the information provided by the two-dimensional (2D) and three-dimensional (3D) approaches. In addition, microglial morphological changes was explored after Aβ immunotherapy, using our unique group of AD patients immunised against Aβ42^10^.

## Results

### 2D-morphological assessment

**Figure 1:**
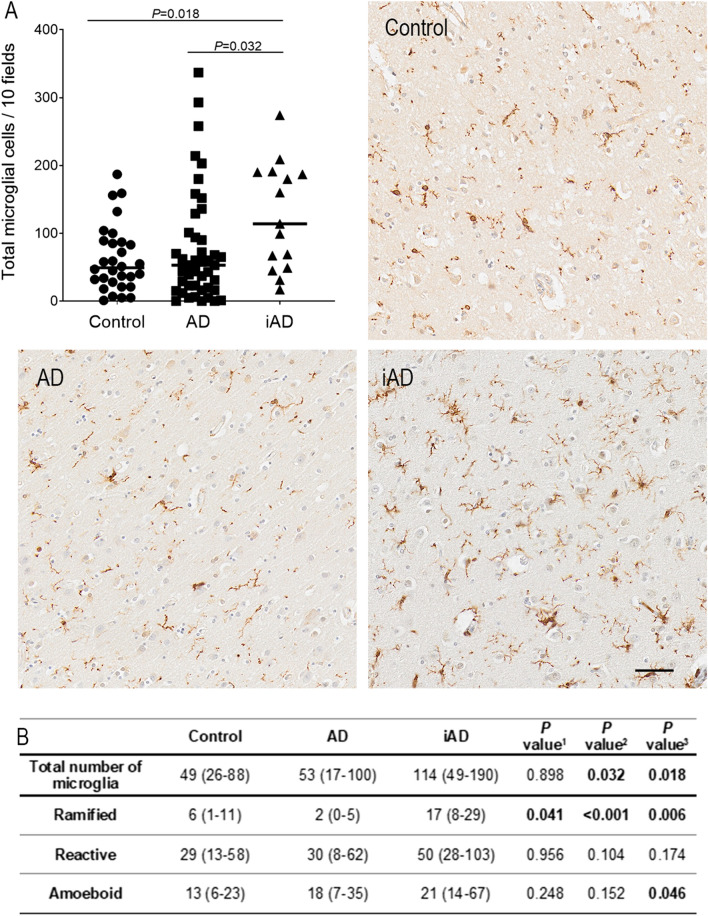
(**A**) Graph and illustration of the number of microglia within the three groups showing increased Iba1 + microglial cells in the iAD group compared to control and AD groups. Haematoxylin counterstaining. Brain area: inferior parietal lobule. Scale bar = 50 µm. (**B**) Quantification of number of microglial cells per 10 fields in 32 controls, 44 AD and 16 iAD cases. Total n = 192 cases. Values in the tables are presented as median with IQR. *P* value by Kruskal–Wallis test with Benjamini–Hochberg correction for multiple testing—*P* value^1^: control vs AD; P value^2^: AD vs iAD. P value^3^: Control vs iAD. Significant *P* value in bold.

Quantification of the total number of microglial cells showed the density of microglia was more than twice as high in the iAD group (median = 114 microglia/10 fields) compared to the AD group (median = 53 microglia/10 fields, *P* = 0.032) and to the control group (median = 49 microglia/10 fields, *P* = 0.018). There was no difference between the control and AD groups (*P* = 0.898) (Fig. 1).

The density of microglia with ramified morphology was lower in the AD group than the control group (*P* = 0.041). However, ramified morphology was substantially increased in the iAD cases (median = 17 ramified cells/10 fields) compared to AD (median = 2 ramified cells/10 fields, *P* < 0.001) and to controls (median = 6 ramified cells/10 fields, *P* = 0.006). The microglial population with reactive morphology was unchanged among the three groups. The number of microglia with amoeboid morphology was not different between controls and AD and between AD and iAD groups. However, comparison of iAD cases with the control group showed the amoeboid microglial population to be modestly increased in iAD (13 vs. 21 amoeboid cells/10 fields, *P* = 0.046) (Fig. 1).

Additionally, we compared the distribution of the proportion of cells of each morphological category. Similarly, the proportion of ramified cells was decreased in AD compared to controls (3.45 vs. 8.54%, *P* = 0.012), while increased more than threefold in iAD compared to AD cases (14.49 vs. 3.45, *P* < 0.001) and nearly twofold compared to controls (14.49 vs. 8.54, *P* = 0.034). There was no significant difference in the proportion of reactive or amoeboid cells among the three groups. However, when pooling ramified and reactive cells together, we observed significant differences reflective of the changes observed in the proportions of ramified microglia. Reactive and amoeboid cells (pooled together) were increased in the AD group compared to controls (96.55 vs 91.46%, *P* = 0.012) and decreased in iAD compared to AD (85.00 vs 96.55%, *P* < 0.001) and to controls (85.00 vs. 94.46%, *P* = 0.034). It is worth noting that, despite the proportion of reactive and amoeboid cells being decreased in the iAD group compared to controls, the actual density of amoeboid cells was increased, due to the overall increase in the density of microglial cells (Table 1).

**Table 1:** Distribution of the proportion of each morphological category (%) in controls, AD and immunised AD groups.

	Control	AD	iAD	*P* value^1^	*P* value^2^	*P* value^3^
Total number of microglia	49 (26–88)	53 (17–100)	114 (49–190)	0.898	**0.032**	**0.018**
Ramified (%)	8.54 (1.94–26.48)	3.45 (0.00–7.06)	14.49 (9.09–25.81)	**0.012**	**< 0.001**	**0.034**
Reactive (%)	57.01 (42.86–67.15)	60 (49.53–66.40)	55.08 (31.88–65.45)	0.707	0.732	0.998
Amoeboid (%)	26.98 (19.36–37.86)	33.33 (25.07–47.78)	29.32 (18.42–38.89)	0.831	0.318	0.186
Reactive + amoeboid (%)	91.46 (73.52–98.06)	96.55 (92.94–100)	85.00 (74.19–90.91)	**0.012**	**< 0.001**	**0.034**

Values are median with IQR.

*P* value by Kruskal–Wallis test with Benjamini–Hochberg correction for multiple testing.

*P* value^1^: control vs. AD; *P* value^2^: AD vs. iAD. *P* value^3^: Control vs. iAD.

Significant *P* value in bold.

We assessed whether the microglial morphology was sex-specific within the three groups. No difference was found between men and women in microglial morphology in the control and or AD groups. However, in the iAD group, women showed an increased proportion of ramified microglia compared to men (women = 28.5%, IQR 15–32 vs. men = 12.0%, IQR 8.5–14.5; *P* = 0.040), and a decreased proportion of reactive microglia (women = 45.5%, IQR 28–50.5 vs. men = 61.7%, IQR 56.5–68.5; *P* = 0.029). It is worth noting that there was no significant difference in the distribution of the frequency of men and women across the study groups (χ^2^ = 2.119; *P* = 0.347).

### 3D assessment of microglial morphological features

Since the 2D assessment revealed significant differences in the distribution of the microglial cells according to their morphological categories, a more detailed assessment of the microglial morphology was performed with the 3D reconstruction of complete individual microglia visualised and reconstructed. The 10 Control and 10 AD cases were selected based on their Aβ load^2^ to ensure that they represent the overall group; whereas, due to the heterogeneity in Aβ removal after Aβ immunotherapy^10^, iAD cases were selected to ensure a representation of the different amyloid changes driven by the treatment.

**Figure 2:**
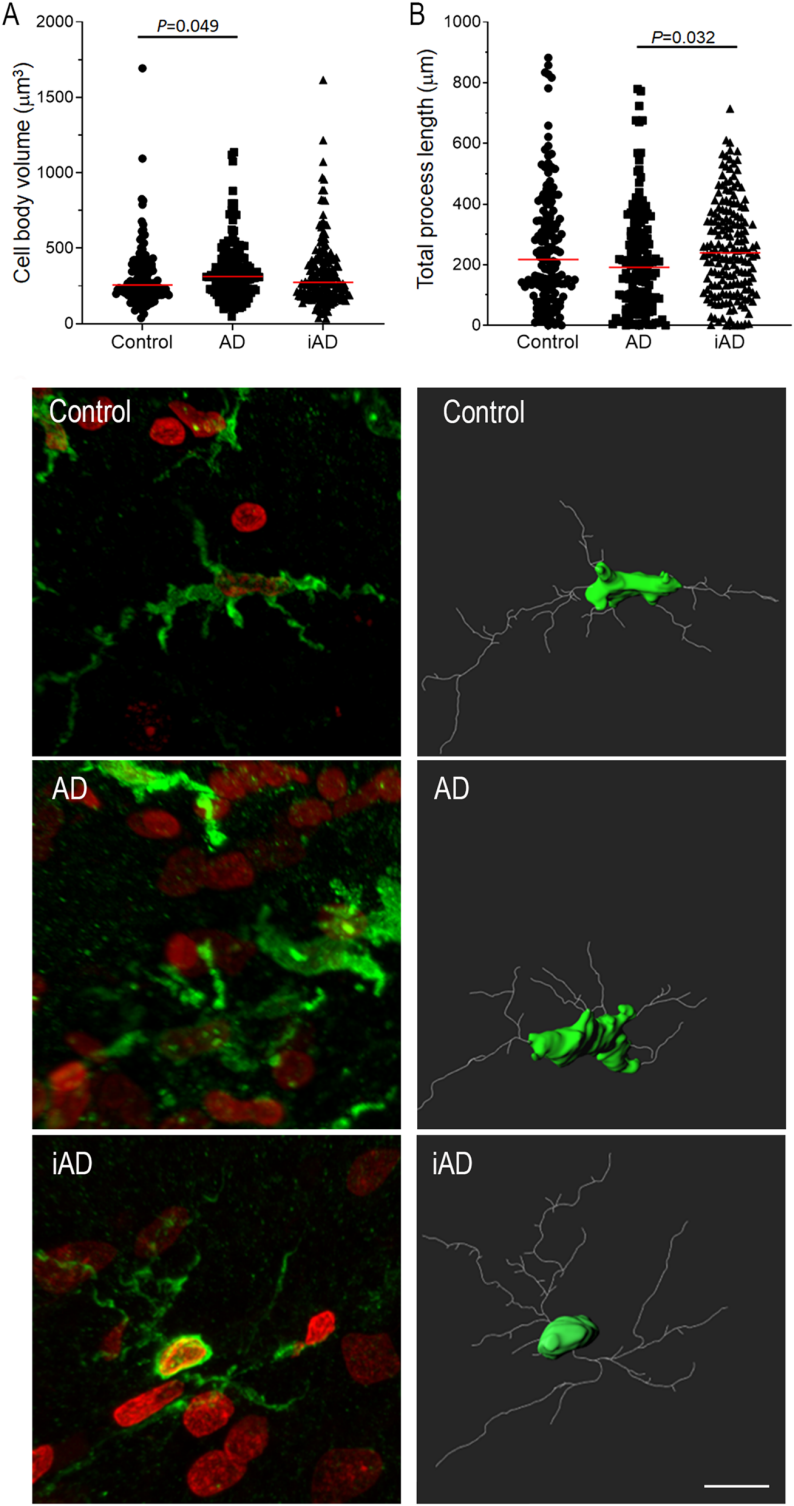
Quantification and illustration of the microglial morphological features after a 3D-reconstruction. (**A**) cell body volume increased in AD (n = 10) compared to controls (n = 10), and (**B**) total process length increased in iAD (n = 15) compared to AD (n = 10). Illustration of microglial cells representative of the median cell body volume and total length for each group. Total n = 35 cases. Left column: maximum intensity projection of the confocal Z-stacks (green—microglial marker Iba1; red—cell nuclei identified with the fluorescent DNA stain DAPI). Right column: 3D-reconstructed models of selected microglia, showing AD with larger body volume than control, and iAD with more extended total process length than AD. Brain area: inferior parietal lobule. Scale bar = 10 μm.

Among the several features measured, the comparison of the morphological features among the three groups shows between the controls and AD cases a significant increase of the cell body volume in AD (313.00 vs. 256.50, *P* = 0.049), and between AD and the iAD groups, a significantly increase of the total process length in the iAD group (238.99 vs. 191.20, *P* = 0.032). Of note, a trend towards increased total primary and secondary process length was observed in iAD compared to AD group (*P* = 0.061 and *P* = 0.067, respectively). No significant difference was found between the three groups for the cell body sphericity, the number of primary processes, the total primary process length, the average primary process length, the average primary process straightness, the total number of junctions, the number of junctions per primary process and the total secondary process length. However, interestingly, no difference was detected between the iAD and the control groups for all the features (Table 2, Fig. 2).

**Table 2:** 3D morphological features of microglia in controls AD and immunised AD cases.

	Control	AD	iAD	*P* value^1^	*P* value^2^	*P* value^3^
Cell body volume (μm^3^)	256.50 (189.00–386.00)	313.00 (219.50–432.00)	274.00 (189.00–421.00)	**0.049**	0.218	0.313
Cell body sphericity	0.652 (0.585–0.705)	0.643 (0.578–0.717)	0.646 (0.583–0.704)	0.985	1.000	1.000
Number of primary processes	6 (4–8)	6 (3–9)	6 (4–9)	0.923	1.000	0.834
Total primary process length (μm)	124.99 (75.67–176.35)	110.84 (60.53–152.45)	128.54 (84.01–169.19)	0.102	0.061	0.544
Average primary process length (μm)	19.56 (13.20–27.14)	17.84 (12.10–24.64)	18.99 (15.12–26.49)	0.114	0.177	0.973
Average primary process straightness	0.757 (0.707–0.801)	0.758 (0.703–0.812)	0.763 (0.717–0.809)	0.631	0.866	0.838
Total number of junctions	21 (11–35)	19 (9–33)	22 (11–36)	0.317	0.290	0.730
Number of junctions per primary process	3.40 (2.00–5.83)	3.00 (1.80–5.29)	3.70 (2.00–5.66)	0.255	0.342	0.887
Total secondary process length (μm)	110.38 (52.66–208.16)	99.34 (37.41–191.32)	129.28 (57.67–217.05)	0.195	0.067	0.483
Total process length (μm)	216.85 (117.32–363.15)	191.20 (83.15–311.22)	238.99 (131.70–359.83)	0.105	**0.032**	0.506

Values are median with IQR.

*P* value by Kruskal–Wallis test with Benjamini–Hochberg correction for multiple testing.

*P* value^1^: control vs AD; *P* value^2^: AD vs iAD. *P* value^3^: Control vs iAD.

Significant *P* value in bold.

### Correlations between microglial morphological features and AD pathology

The relationship was explored between the microglial morphological features measured and the levels of pan-Aβ, Aβ42 and ptau previously quantified and published^2^ to assess whether changes in the morphology reflected protein deposition and pathological changes.

Overall, two negative correlations were detected for pan-Aβ load with total process length in AD cases (r_s_ = − 0.829, *P* < 0.001) and with number of primary processes in iAD cases (r_s_ = − 0.794, *P* = 0.043). No correlation was detected with Aβ42 and ptau (Table 3).

**Table 3:** Correlation of microglial morphological features with pan-Aβ, Aβ42 and ptau.

		Pan-Aβ	Aβ42	ptau			Pan-Aβ	Aβ42	ptau
Cell body volume (μm^3^)	Ctrl	r_s_ = − 0.333 P = 0.751	r_s_ = − 0.188 P = 0.938	r_s_ = − 0.433 P = 0.607	Average primary process straightness	Ctrl	r_s_ = 0.430 P = 0.607	r_s_ = 0.370 P = 0.676	r_s_ = − 0.061 P = 1.000
	AD	r_s_ = − 0.224 P = 0.761	r_s_ = − 0.167 P = 0.877	r_s_ = 0.018 P = 0.979		AD	r_s_ = 0.139 P = 0.892	r_s_ = − 0.283 P = 0.705	r_s_ = 0.152 P = 0.871
	iAD	r_s_ = 0.165 P = 0.919	r_s_ = − 0.021 P = 0.982	r_s_ = − 0.036 P = 1.000		iAD	r_s_ = − 0.165 P = 0.919	r_s_ = 0.146 P = 0.905	r_s_ = − 0.036 P = 1.000
Cell body sphericity	Ctrl	r_s_ = 0.055 P = 0.984	r_s_ = 0.079 P = 0.973	r_s_ = 0.006 P = 0.987	Number of primary processes	Ctrl	r_s_ = − 0.055 P = 0.984	r_s_ = 0.115 P = 0.980	r_s_ = − 0.043 P = 0.987
	AD	r_s_ = 0.588 P = 0.263	r_s_ = 0.017 P = 0.980	r_s_ = 0.673 P = 0.139		AD	r_s_ = − 0.455 P = 0.427	r_s_ = − 0.283 P = 0.705	r_s_ = − 0.103 P = 0.917
	iAD	r_s_ = 0.257 P = 0.757	r_s_ = 0.161 P = 0.923	r_s_ = − 0.327 P = 0.728		iAD	**r**_**s**_** = **− **0.829** **P < 0.001**	r_s_ = − 0.525 P = 0.264	r_s_ = − 0.100 P = 0.957
Total secondary process length (μm)	Ctrl	r_s_ = − 0.018 P = 0.974	r_s_ = 0.176 P = 0.947	r_s_ = − 0.427 P = 0.613	Total number of junctions	Ctrl	r_s_ = − 0.115 P = 0.980	r_s_ = 0.115 P = 0.980	r_s_ = − 0.421 P = 0.624
	AD	r_s_ = − 0.709 P = 0.113	r_s_ = − 0.583 P = 0.310	r_s_ = − 0.539 P = 0.315		AD	r_s_ = − 0.745 P = 0.080	r_s_ = − 0.567 P = 0.322	r_s_ = − 0.588 P = 0.263
	iAD	r_s_ = − 0.411 P = 0.445	r_s_ = − 0.464 P = 0.321	r_s_ = − 0.182 P = 0.909		iAD	r_s_ = − 0.521 P = 0.280	r_s_ = − 0.468 P = 0.339	r_s_ = − 0.155 P = 0.922
Average primary process length (μm)	Ctrl	r_s_ = 0.018 P = 0.974	r_s_ = − 0.055 P = 0.984	r_s_ = − 0.189 P = 0.942	Number of junctions per primary process	Ctrl	r_s_ = − 0.418 P = 0.617	r_s_ = 0.127 P = 0.971	r_s_ = − 0.439 P = 0.603
	AD	r_s_ = − 0.527 P = 0.328	r_s_ = − 0.317 P = 0.682	r_s_ = − 0.358 P = 0.587		AD	r_s_ = − 0.600 P = 0.251	r_s_ = − 0.317 P = 0.682	r_s_ = − 0.673 P = 0.139
	iAD	r_s_ = 0.121 P = 0.899	r_s_ = − 0.161 P = 0.923	r_s_ = − 0.091 P = 0.970		iAD	r_s_ = − 0.156 P = 0.904	r_s_ = − 0.293 P = 0.698	r_s_ = − 0.264 P = 0.798
Total primary process length (μm)	Ctrl	r_s_ = 0.224 P = 0.917	r_s_ = 0.248 P = 0.934	r_s_ = 0.030 P = 1.000	Total process length (μm)	Ctrl	r_s_ = 0.030 P = 0.991	r_s_ = − 0.079 P = 0.973	r_s_ = − 0.128 P = 0.975
	AD	r_s_ = − 0.733 P = 0.093	r_s_ = − 0.067 P = 0.956	r_s_ = − 0.285 P = 0.681		AD	**r**_**s**_** = **− **0.794** **P = 0.043**	r_s_ = − 0.400 P = 0.567	r_s_ = − 0.418 P = 0.486
	iAD	r_s_ = − 0.516 P = 0.059	r_s_ = − 0.461 P = 0.084	r_s_ = − 0.082 P = 0.968		iAD	r_s_ = − 0.503 P = 0.306	r_s_ = − 0.514 P = 0.276	r_s_ = − 0.100 P = 0.957

r_s_: Spearman’s rank correlation coefficient.

*P* values adjusted by Benjamini–Hochberg correction for multiple testing.

Significant values in bold.

*Ctrl * Control group.

To explore further the associations, a different approach was used. Within each study group, cases were grouped according to their pan-Aβ load, using the median of each group as a cut-point. Then the morphological features were compared between the low Aβ and high Aβ groups, to investigate whether the load of Aβ affects microglial morphology (Table 4).

**Table 4:** Comparison of morphological features between low and high Aβ cases.

		Low Aβ	High Aβ	*P* value			Low Aβ	High Aβ	*P* value
Cell body volume (μm^3^)	Ctrl	295.5 (221.8–421.5)	237.0 (182.8–375.3)	0.054	Average primary process straightness (μm)	Ctrl	0.760 (0.689–0.807)	0.754 (0.708–0.798)	0.879
	AD	318.0 (232.0–431.0)	302.0 (186.0–438.0)	0.274		AD	0.766 (0.704–0.817)	0.754 (0.701–0.810)	0.525
	iAD	263.0 (192.0–384.0)	298.5 (184.0–478.0)	0.218		iAD	0.772 (0.720–0.811)	0.758 (0.716–0.806)	0.445
Cell body sphericity	Ctrl	0.661 (0.580–0.716)	0.650 (0.585–0.701)	0.904	Number of primary processes	Ctrl	6.0 (4.8–8.0)	6.0 (4.0–8.0)	0.456
	AD	0.621 (0.561–0.690)	0.676 (0.620–0.752)	0.272		AD	7.0 (4.0–9.0)	5.0 (3.0–8.0)	**0.008 (0.71)**
	iAD	0.641 (0.583–0.702)	0.651 (0.579–0.721)	0.721		iAD	7.0 (5.0–9.0)	5.0 (4.0–8.0)	**0.003 (0.71)**
Total secondary process length (μm)	Ctrl	190.6 (86.2–322.5)	105.9 (48.0–179.5)	**0.011 (0.56)**	Total number of junctions	Ctrl	26.0 (15.3–47.8)	20.0 (10.3–32.8)	**0.019 (0.77)**
	AD	130.7 (57.9–223.6)	69.2 (23.1–139.5)	**0.001 (0.53)**		AD	24.0 (14.0–38.0)	14.0 (6.0–25.0)	**< 0.001 (0.58)**
	iAD	135.7 (72.0–237.9)	106.2 (41.9–205.4)	**0.048 (0.78)**		iAD	25.0 (14.0–39.0)	18.5 (9.0–34.0)	**0.017 (0.74)**
Average primary process length (μm)	Ctrl	25.9 (15.6–30.2)	18.9 (12.9–26.0)	**0.033 (0.73)**	Number of junctions per primary process	Ctrl	5.4 (2.0–7.3)	3.3 (2.0–5.3)	0.052
	AD	17.2 (12.7–25.8)	19.4 (10.6–24.1)	0.779		AD	3.8 (2.0–6.2)	2.3 (1.6–4.8)	**0.017 (0.61)**
	iAD	18.6 (15.4–24.4)	20.3 (14.8–27.0)	0.519		iAD	3.8 (2.2–5.7)	3.4 (1.9–5.6)	0.056
Total primary process length (μm)	Ctrl	146.0 (81.5–196.6)	118.6 (71.8–162.6)	0.121	Total process length (μm)	Ctrl	339.9 (132.9–475.8)	187.8 (102.0–324.9)	**0.015 (0.55)**
	AD	129.6 (78.7–169.9)	98.1 (51.0–130.7)	**0.005 (0.76)**		AD	219.1 (111.1–380.3)	156.3 (70.5–258.6)	**0.003 (0.71)**
	iAD	132.5 (98.0–169.2)	123.7 (67.0–170.1)	0.185		iAD	259.6 (144.9–402.5)	216.8 (103.1–333.0)	**0.031 (0.84)**

Values are median with IQR.

*P* value by the independent samples Mann–Whitney U test (low Aβ vs high Aβ within each group).

Significant *P* values in bold with fold change (high Aβ with respect to low Aβ) between brackets.

*Ctrl* Control group.

In controls, a high load of Aβ was associated with reduced total secondary process length (105.9 vs. 190.6 µm, *P* = 0.011), reduced average primary process length (18.9 vs. 25.9 µm, *P* = 0.033), reduced total number of junctions (20 vs. 26, *P* = 0.019), and reduced total process length (187.8 vs. 339.9 µm, *P* = 0.015).

In AD, a high Aβ load was associated with reduced total secondary process length (69.2 vs. 130.7 µm, *P* = 0.001), reduced total primary process length (98.1 vs. 129.6 µm, *P* = 0.005), reduced number of primary processes (5 vs. 7, *P* = 0.008), reduced total number of junctions (14 vs. 24, *P* < 0.001), reduced number of junctions per primary process (2.3 vs. 3.8, *P* = 0.017), and reduced total process length (156.3 vs. 219.1, *P* = 0.003).

Lastly, in the iAD group, higher levels of Aβ were associated with reduced total secondary process length (106.2 vs. 13,537 µm, *P* = 0.048), reduced number of primary processes (5 vs. 7, *P* = 0.003), reduced total number of junctions (2.3 vs. 3.8, *P* = 0.017), and reduced total process length (216.8 vs. 259.6 µm, *P* = 0.031).

## Discussion

The present study focuses on evaluating microglial morphology using 2D and 3D approaches. The marker Iba1 was chosen as optimal for this purpose, as its expression reflects microglial motility^11^, a key function of microglia, while at the same time allowing visualisation of the cell morphology with a reliable degree of detail. Here, we show that in the brain of aged humans, the most common microglial morphology was the reactive followed by the amoeboid, while the ramified microglia were the least frequent morphology. Interestingly, this was observed in the three groups studied, despite showing significant differences in the amounts of cells within each category. This observation is of particular interest if we compare the human findings with the frequency of morphologies observed in the mouse brain, in which around 90% of microglial cells are ramified, using similar morphological criteria^4^, versus 9% in the aged controls in our study. This striking difference between mice and humans is likely evidence of the impact of the environment in which mice and humans live and differences in their respective lifespans. Laboratory mice typically live in a controlled, relatively pathogen-free environment, while humans are normally exposed to a broad range of pathogens throughout life, which activate microglia, inducing a change toward a reactive or amoeboid morphology. The difference in lifespan between mice and humans could also have an influence in the morphologies observed. Ageing in humans is commonly associated with cerebrovascular pathology and the effects of transient or irreversible ischaemia which may activate microglia. It was reported that human microglia have an average lifespan of 4.2 years^12^, which exceeds the average life expectancy of most laboratory mice. A longer lifespan will be associated with a longer time of exposure with the environment and thus increases the likelihood of microglia to become primed and retaining a memory of past events^13^. These considerations may have clinical implications in translational research, especially in the context of manipulating microglia for therapeutic purposes.

The high frequency in which reactive and amoeboid cells are present in the human brain, even in the control group, is noteworthy considering the way the ramified form is commonly referred to as “homeostatic”, implying the presence of reactive/amoeboid cells is pathological. However, this might not necessarily be indicative of a pathological condition. In fact, reactive/amoeboid microglia perform a directed motility function, also known as chemotactic motility, which consists in a targeted extension of processed towards the source of injury in order to restore the homeostasis^11^. Therefore reactive/amoeboid microglia may be also important for the health of the brain. Consequently, an imbalance in the proportion of ramified *vs.* reactive/amoeboid cells may be the important factor associated with any neuropathological condition with an inflammatory reaction. However, the 2D measurement might underestimate the true numbers as features above and below the section are not observed (e.g. volume, processes length, ramifications).

The 3D analysis of microglia provided novel important additional information of human microglia in human aged brain which included a cell body sphericity not very spherical and an average of 6 primary processes, with 3–4 junctions per process. Interestingly, these morphological features were highly similar between each group, highlighting intrinsic features of microglia, but also very different from mouse microglia with a study identifying an average of 167 of junctions per microglia in wild-type mice^7^, emphasizing striking difference between the highly ramified microglia in mouse and the more reactive/amoeboid morphology observed in humans.

In AD, we did not observe a difference in the number of microglia compared to controls, consistent with our previous findings assessing the expression of Iba1 expression as protein load^2^, and as reported in smaller cohorts. Indeed, these human post-mortem studies did not provide evidence of increased microglial numbers in AD, with one study even reporting less microglial cells associated with the disease^8,9^. However, our observation is in contrast with most studies in AD mice models, in which increased numbers of microglia are typically found in the hippocampus and the cerebral cortex^14,15^. This discrepancy between the experimental models and our findings might be of importance considering there are currently therapeutic approaches developed based on the idea of microglial proliferation in AD^16–19^. It is noteworthy that similar to the unchanged Iba1 expression in AD in our current study, we recently described in the same cohort, unmodified expression of other motility-related microglial proteins P2RY12, coronin-1A and cofilin-1^2^.

Our results point to some important changes in microglial morphology, relevant to AD. The 2D assessment highlighted a reduction in ramified microglial population to approximately a third compared to controls, consistent with previous publication in a smaller cohort^8^. Consequently, we were expecting a decrease in the morphological features directly associated with ramification such as number of junctions or total process length, but this was not observed. In addition, the 3D-morphological assessment showed increased cell body volume in AD; which according to the morphological classification is part of the criteria to classify the cell as “reactive”, a cell population which was not different between controls and AD. However, the morphological changes noticed in our study are relevant to the increased microglial activation that we reported in AD using CD68 (phagocytic activity), macrophage scavenger receptor (MSR)-A (scavenging activity), and CD64 and CD16 (Fcγ receptor I and III respectively, and central effectors of immunoglobulins mediated immune response)^20–22^.

The comparison between the 2D and 3D assessment points out the limitations of the 2D methodology. The two previous studies on human microglial morphology in AD reported a reduced total “branch” length and a reduced total number of junctions^9^, or total number of branches^8^. However, it is important to consider that there are significant differences in their methodology compared to the present study including the definition of “branch” that does not distinguish between primary and secondary processes, and the small sample size explored with 16 cells compared to our 525 cell analysis.

The changes in microglial morphology observed after Aβ immunotherapy highlighted several interesting effects of the treatment on microglia. The total number of microglia was twice the number found in both AD and control groups, with various possible explanations which can be summarised as: (i) induction of microglial proliferation, (ii) enhanced Iba1 expression by pre-existing microglia, or/and (iii) recruitment of peripheral monocytes/macrophages. Regarding microglial proliferation, it is known that microglial density in the brain remains stable throughout the lifespan. This stability is achieved by a self-renewal process characterised by a coupled mechanism of proliferation and apoptosis^23^, and it could be hypothesised that immunotherapy promotes microglial proliferation, although the mechanism of this is unknown and proliferation due to immunotherapy remains to be confirmed. Interestingly, the marker VEGF, previously reported increased after Aβ42 immunotherapy^2^, can induce microglial proliferation and promote directed motility in vitro, suggesting the possibility of a link between these two phenomena occurring after immunotherapy^24^. It is also possible that the observed increase in the total number of microglia does not reflect proliferation but simply increased Iba1 expression per cell. So far, Iba1 is considered as a pan-microglial marker with the assumption that it is expressed by all microglial cells. However, this view has been recently challenged by new evidence of CD68 or P2RY12-positive microglia that are Iba1-negative^25,26^. This means that the augmentation in Iba1-positive microglia does not necessarily imply a true increase in the number of cells, but rather could be interpreted as an increase in microglial motility, considering the role of Iba1 as an actin-related cytoskeletal protein^11,27^, and in accordance with the reported elevated purinergic receptor P2RY12 expression in this cohort^2^. Additionally, recruitment of macrophages from the periphery could be a source for the additional Iba1-positive cells observed^28^.

After Aβ immunotherapy, the ramified population was increased twofold compared to AD, and the detailed 3D assessment of the morphological features revealed increased total process length after the immunotherapy. This increase in ramified microglia, regarded as “homeostatic”, is consistent with the overall downregulation of microglial activation we observed in a previous study with the markers CD68, MSR-A, CD64 (FcgRI) and CD32 (FcgRII) when Aβ is removed by immunotherapy^29,30^. With a higher total number of Iba1-positive microglia, and the cells having longer processes, these findings indicate that a much greater volume of the brain parenchyma is being surveyed by microglia. Therefore, the immunotherapy appears to engage microglia towards parenchyma surveillance defined as baseline motility in physiological conditions^11^; though the consequences on the brain function are unknown. Of note, the immunotherapy seems to enhance the ramified population in women which could point out a sex-associated difference in the microglial response to the immunotherapy treatment, although the number available for analysis was small. However, the morphological changes observed after immunotherapy confirms that the treatment does not restore the profile observed in the healthy brain^2^.

The investigation of the relationship between the microglial morphological features and AD pathology showed that increased Aβ accumulation in the parenchyma is associated with decreased total process length of microglia. This is consistent with the increased reactive/amoeboid cell population reported with the 2D assessment in AD, the cognitive decline associated with phagocytic microglia (CD68, MSR-A, HLA-DR, CD64)^21^ and the sequence of events recently proposed to explain AD neuropathogenesis in which the microglial reaction to Aβ is a key step^31^. Microglia react to the presence of Aβ in the parenchyma by shortening their processes. When Aβ has been removed by immunotherapy, this leads to an increased number of primary processes, consistent with the increased ramified population reported here, increased Iba1 and P2RY12 as markers of homeostatic microglia2 and the overall microglial downregulation previously reported in this cohort^29,30^.

Our second analysis of the microglial morphology according to Aβ load stressed the effect of Aβ on microglia. Indeed, even though the specific associations vary from one group to another, all of them were consistent with high Aβ load related to a decrease in measurements of process length and/or branching in the three groups. Similar findings were reported in another human study using a 2D assessment, with the higher pathology cases (defined by their ABC scores) having increased number of “activated” microglia (microglia with reactive or amoeboid morphology, i.e. with less ramifications). They also observed an inverse correlation between total “branch” length and Aβ^8^. Of note, the addition of our immunised cases allowed us to observe a beneficial aspect of the treatment with microglia in the immunised patients presenting a morphology similar to old age controls.

In our study, we focused on evaluating the detailed morphological features of individual microglia. While the morphology of microglia clustering around plaques has been assessed in experimental models^7^, this was not feasible in our human cohorts for two reasons. Firstly, clustering of microglia around plaques complicates the identification of individual processes, and secondly, the high variability in amyloid plaques in the iAD cohorts, with some of them nearly completely cleared of Aβ^10^, will exclude some cases. In addition, it has been estimated that only 2% of microglia in AD are clustered in association with plaques, conversely the vast majority are not^8^. Therefore, to ensure consistency and reproducibility of the assessment between our cohorts, we selected individual microglia cells at random, regardless of proximity to plaques. Nevertheless, the associations with overall Aβ load were detected even though the cells analysed were not specified as plaque-associated.

To conclude, the present study constitutes a detailed assessment of the microglial morphology in the human brain, analysing microglia in the context of AD and after Aβ-immunotherapy. This assessment has been done coupling different approaches, including cell quantification, morphological classification, and a detailed measurement of several morphological features by the use of semi-automated methods of 3D-reconstruction. Interestingly, our study supports that 2D and 3D assessments provide different but consistent information on microglial morphology. We showed that in the aged human brain, reactive/amoeboid microglia are the most numerous population, consistent with the priming effect due to age and environment. In AD, the number of microglia is not affected but the ramified population is decreased towards the reactive/ramified population as the response to Aβ accumulation; whereas Aβ immunisation led to an increase in the ramified microglial population, higher than in controls. Via our unique cohort of immunised AD cases, our 3D assessment underlined that even in an old brain affected by AD pathology for several years, microglia retain a certain morphological plasticity with the potential consequences remaining to be investigated.

## Methods

### Cases

Brain tissue samples from 76 donors were sourced from the South West Dementia Brain Bank, comprising 44 AD cases and 32 controls. AD cases had a clinical diagnosis of sporadic AD made during life and satisfied post-mortem neuropathological consensus criteria for AD^32^. Cases with any other significant brain pathologies such as stroke, tumour or traumatic brain injury were excluded from the study. Controls were aged-matched cases, with no history of neurological or psychiatric disease or symptoms of cognitive impairment. Additionally, samples from 16 AD patients who participated in the first AN1792 clinical trial of Aβ immunotherapy (iAD) were also included in the study. Paraffin sections (4 µm thickness) of formalin-fixed brain tissue from the inferior parietal lobule, as a region of cerebral cortex affected by AD pathology^32^, were slices studied.

Thicker slices (50 µm thickness) were selected from 10 controls, 10 AD cases and 15 iAD cases for a more detailed 3D assessment of morphological features. A summary of the demographic, clinical and post-mortem characteristics of the groups can be found in Table 5.

**Table 5:** Demographic, clinical and post-mortem characteristics of the three groups. *Control* neurologically/cognitively normal controls, *AD* Alzheimer’s disease cases, *iAD* immunised Alzheimer’s disease cases *F* female, *M* male, *APOE* genotyping was not available for all cases *n/a* not-applicable, S*D* standard deviation.

Groups	Control n = 32	AD n = 44	iAD n = 16
Gender	17F:15M	28F:16M	7F:9M
Age of death (years, mean ± SD)	84 ± 7	80 ± 6	79 ± 8
Age of AD onset (years, mean ± SD)	n/a	70 ± 7	67 ± 8
Duration of AD (years, mean ± SD)	n/a	10 ± 3	12 ± 4
Braak stage	0–II: 29	0–II: 0	0–II: 0
	III–IV: 3	III–IV: 4	III–IV: 1
	V–VI: 0	V–VI: 40	V–VI: 15
***APOE* genotype**			
*ε*4*/–*	3/28 (10.7%)	13/38 (34.2%)	6/10 (60.0%)
*ε*4/*ε*4	1/28 (3.6%)	9/38 (23.7%)	3/10 (30.0%)
Post-mortem delay (hours, mean ± SD)	42 ± 23	42 ± 26	22 ± 25

### Ethics approval

We confirmed that all methods were carried out in accordance with relevant guidelines and regulations. The study was covered by the following ethical approvals: (i) the South West Dementia Brain bank (NRES Committee South West Central Bristol, REC reference: 08/H0106/28 + 5 for the controls and AD cases; (ii) the Southampton and South West Hampshire Local Research Ethics Committees (reference: LRC 075/03/w) for the iAD cases.

### DAB-Immunohistochemistry and 2D-morphological assessment

3,3'-Diaminobenzidine (DAB)-immunohistochemistry was performed on the 4 µm-thick sections, using the microglial marker Iba1 (rabbit polyclonal, Wako Chemicals) at a concentration of 1:750. Bound secondary antibody was visualized using the avidin–biotin–peroxidase complex method (Vectastain Elite, Vector Laboratories) with DAB as chromogen and 0.05% hydrogen peroxide as substrate (Vector Laboratories). All sections were counterstained with haematoxylin, then dehydrated and mounted in Pertex (Histolab Products AB). The staining was performed in two batches with each batch containing cases from all groups (Control, AD, iAD). All experiments included a negative control slide incubated in buffer with no primary antibody and a positive control slide containing human tonsil, a tissue type known to express Iba1.

**Figure 3:**
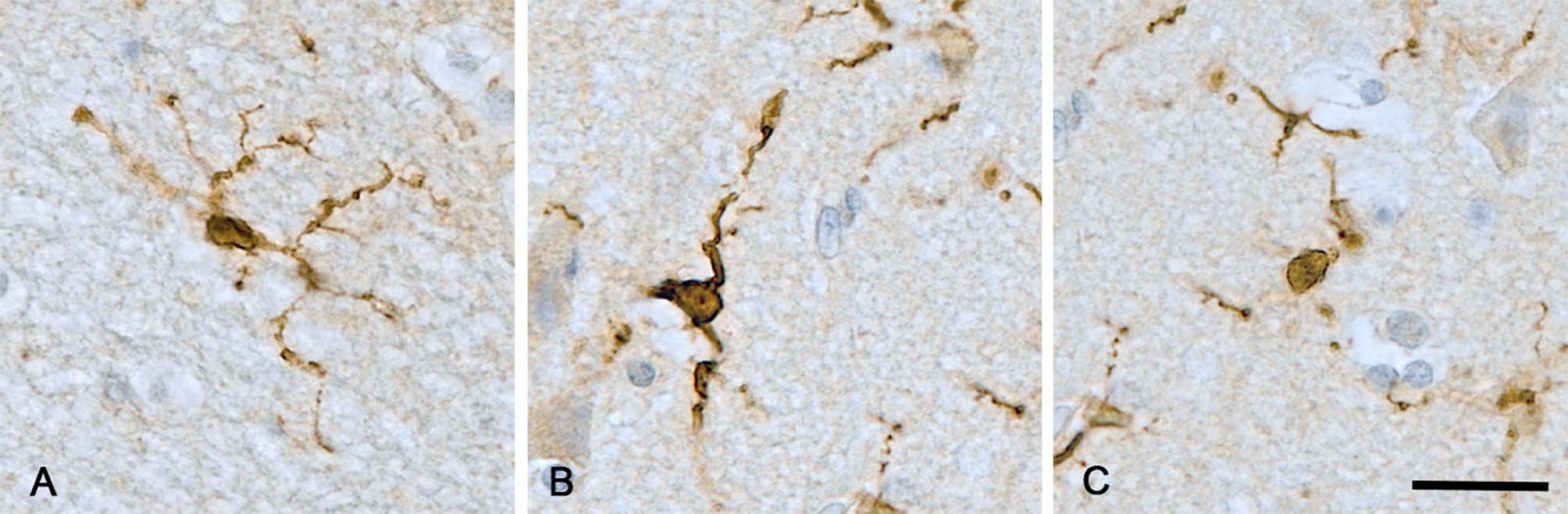
Illustration of the criteria used for the 2D morphological assessment of Iba1-positive microglia showing (**A**) ramified; (**B**) reactive and (**C**) amoeboid microglial cell. Haematoxylin counterstaining. Brain area: inferior parietal lobule. Scale bar = 20 μm.

For the 2D morphological evaluation of microglia, for each case, a digital image was acquired at magnification × 20 with the automated slide scanner microscope Olympus VS110 v2.9.1 (Olympus America Inc., www.olympus-sis.com) and 10 regions of interest (ROIs of 0.25 mm^2^ each) extracted from the grey matter with the Olympus VS-Desktop software. A total of 920 images were analysed as follows: Microglial cells in each ROI were quantified and classified into a morphological category modified from Ref.^4^. Criteria for the three categories considered are (Fig. 3):

Ramified microglia: small cell body; presence of 4 or more long, thin, highly branched processes (Fig. 3A).

Reactive microglia: increased cell body size, reduced number of processes (2–3), processes appear unbranched or with fewer, smaller branches (Fig. 3B).

Amoeboid microglia: increased cell body size, with no processes or 1–2 shortened and unbranched processes (Fig. 3C).

### Fluorescent immunohistochemistry and confocal microscopy

Fluorescent immunohistochemistry was performed on the 50 μm-thick sections using the same anti-Iba1 antibody at a concentration of 1:500. Slides were incubated overnight at room temperature and the next day re-incubated with a second dose of the primary antibody, followed by a secondary Alexa fluor 635 goat anti-rabbit antibody (Thermofisher Scientific) at a concentration of 1:100. The slides were then incubated in a 0.5% Sudan Black B solution to eliminate autofluorescence. As a counterstain, cell nuclei were stained with 4′,6-diamidino-2-phenylindole (DAPI) (1:75 dilution) and mounted with Mowiol mounting medium (Sigma-Aldrich).

The stained slides were examined under a confocal microscope (Leica TCS SP8). Using Leica LASX (Leica Application Suite X) software v3.6.0.20104 (www.leica-microsystems.com), five Z-stacks (each one composed of 100 focal planes) were taken from the grey matter of each of the sections under a × 63 objective.

### 3D-Reconstruction and assessment of morphological features

**Figure 4:**
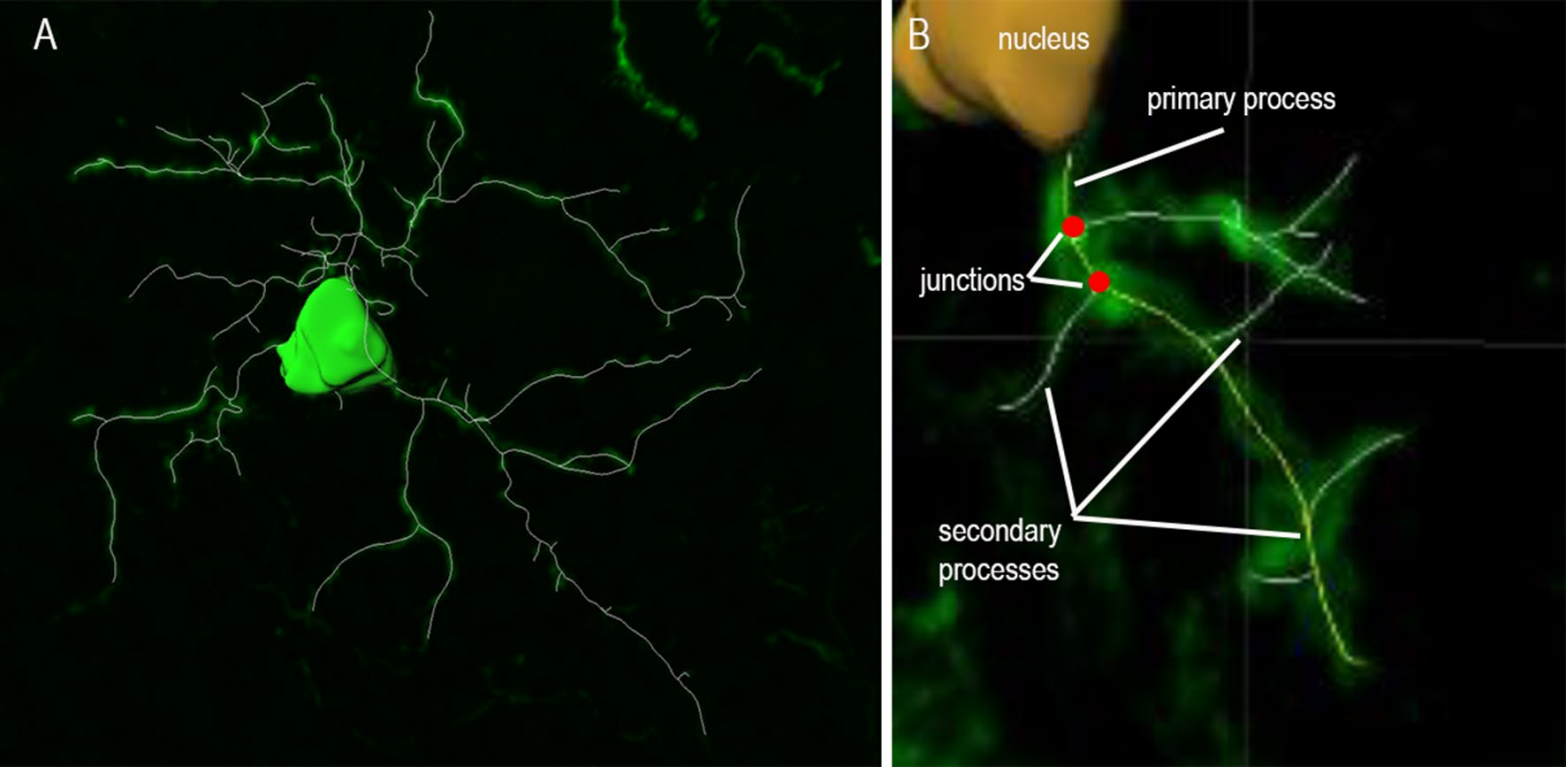
Example of microglial cell after 3D-reconstruction in Imaris with illustration of (**A**) primary processes, (**B**) junctions and (**C**) secondary processes.

Per case, 15 individual microglia were identified from the grey matter at random, regardless of proximity to plaques, and reconstructed in 3D using Imaris (Bitplane) software v7.6 (Oxford Instruments, UK; www.imaris.com). Within each Z-stack, the three microglial cells most centrally located in the XY perspective of the stack and with a complete cell nucleus, as observed by the DAPI signal, were selected for 3D-reconstruction. The cell bodies were constructed using the Imaris function “Surfaces”, with which the contour of the cell body is manually drawn for each focal plane (Fig. 4A). Once all planes the cell body occupies are drawn, the function “Create Surface” automatically turns the contours drawn into a 3D-object. The microglial processes were then semi-automatically traced using the Imaris functions “AutoPath” and “AutoDepth” (Fig. 4A). The 3D-reconstructed model was then used to measure the following morphological features: cell body volume, cell body sphericity (defined as the ratio of the surface area of a sphere with the same volume as the cell body, to the actual surface area of the cell body), number of primary processes (defined as the longest filament segments projecting straight from the cell body), total primary process length, average primary process length, average primary process straightness (defined as the ratio of the distance between the start point to the end point of each primary process to the actual primary process length), total number of junctions (defined as the points where processes divide to project ramifications) (Fig. 4B), number of junctions per primary process, total secondary process length (defined as the total length of all filament segments not directly connected to the cell body), and total process length (defined as the total sum of the length of the primary processes and the secondary processes of each cell).

### Previous findings

Pan-Aβ, Aβ42 and hyperphosphorylated (p)tau protein loads previously obtained from these three groups and published^2^ were used to assess the potential relationships between the 3D morphological features of microglia and AD pathology.

### Statistical analyses

Statistical analyses were performed with the statistical software IBM SPSS v24 (SPSS Inc. Chicago IL). The normality of distribution of each variable considered was assessed by the Shapiro–Wilk test and the homogeneity of variances by the Levene test. When making single-variable comparisons among the study groups, if the variable met the assumptions of normality and homogeneity of variance, the parametric test ANOVA was used. If assumptions were not met, the non-parametric Kruskal–Wallis test was used. Correlations between the 3D microglial features and AD pathology were tested with the non-parametric Spearman’s rank correlation test, as most variable distributions were non-parametric. To account for multiple testing, the Benjamini–Hochberg procedure to control for the false discovery rate (FDR) was used as post-hoc correction in all tests. For all cases, an adjusted *P* value < 0.05 was considered significant.

## Data Availability

The data used and/or analysed during the current study are available from the corresponding author on reasonable request.
